# Supplementary material for: Large-scale genome-wide analysis links lactic acid bacteria from food with the gut microbiome
Source: Nat Commun. 2020 May 25;11:2610. doi: 10.1038/s41467-020-16438-8 (PMC7248083; doi:10.1038/s41467-020-16438-8)
Supplement: Supplementary file 1 — Supplementary Information [file 41467_2020_16438_MOESM1_ESM.pdf]

Large-scale genome-wide analysis of lactic acid bacteria bridges food and gut microbiome

Pasolli *et al.*

Supplementary Information

Supplementary Figures

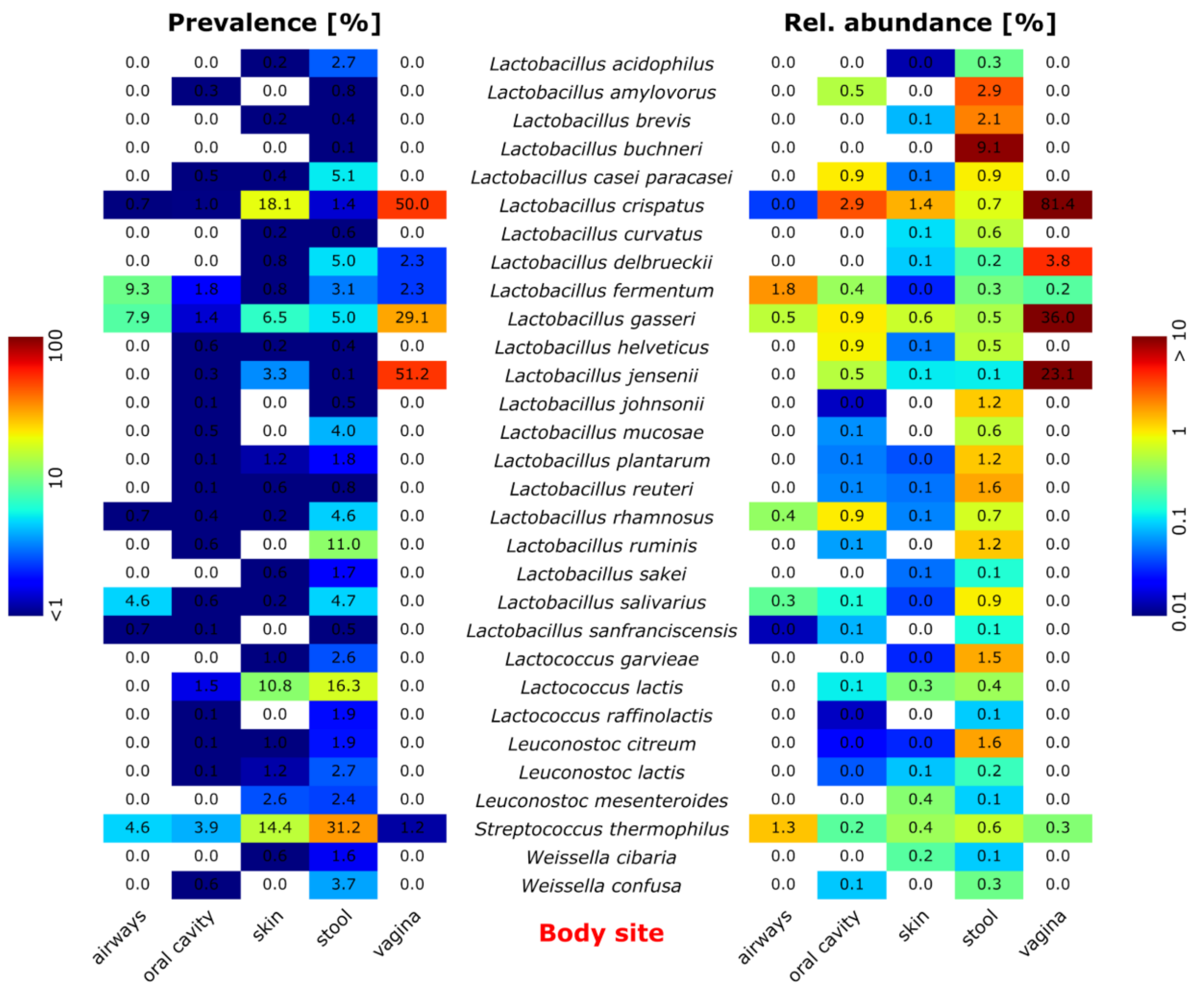

Supplementary Figure 1. Average prevalence and relative abundance of LAB species from the human microbiome stratified by body site. Average relative abundance is computed on positive samples only. Raw data in **Supplementary Data 2**.

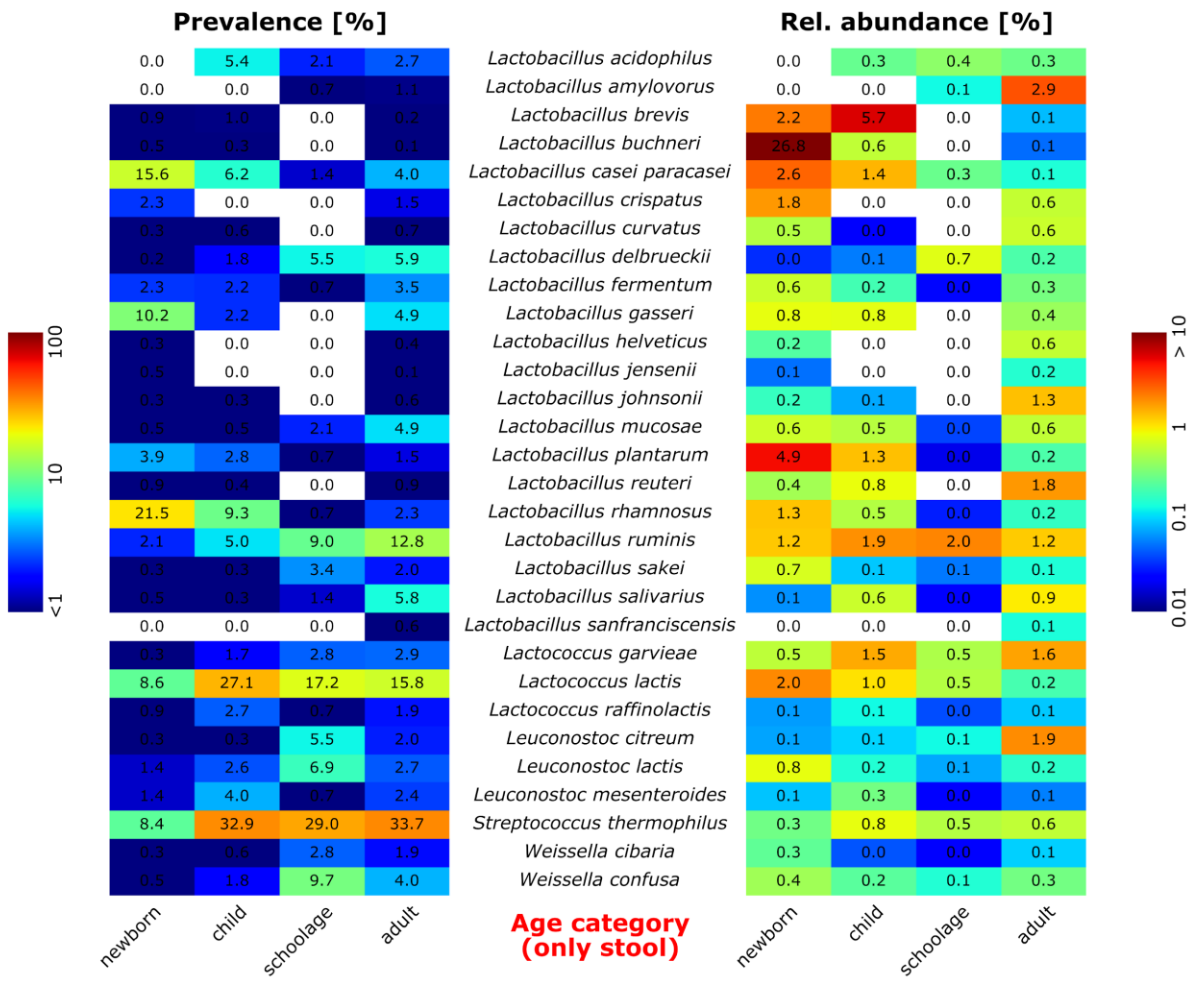

Supplementary Figure 2. Average prevalence and relative abundance of LAB species from the human microbiome stratified by age category. Statistics refer to stool samples only. Average relative abundance is computed on positive samples only. Raw data in **Supplementary Data 2**.

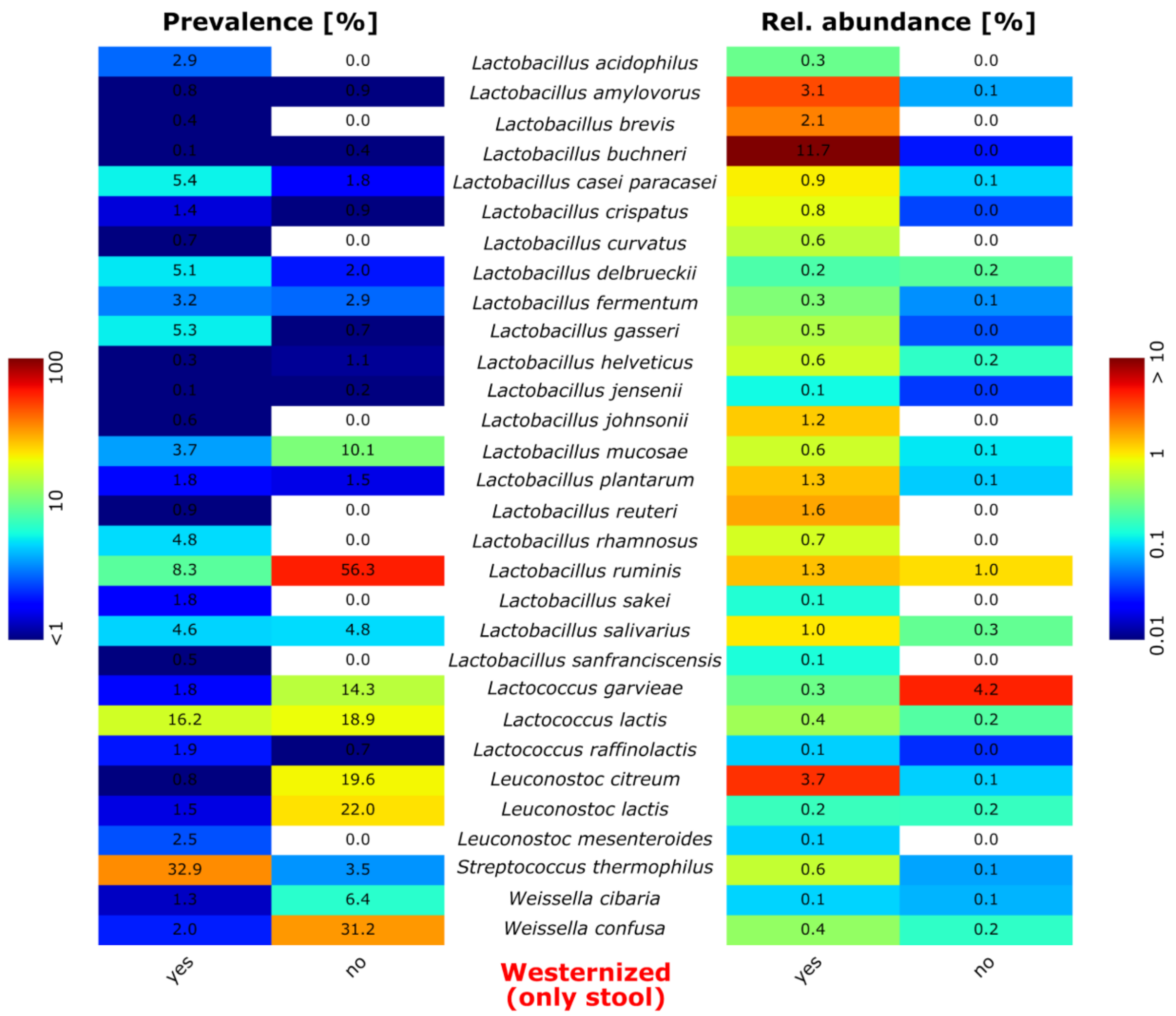

Supplementary Figure 3. Average prevalence and relative abundance of LAB species from the human microbiome stratified by westernized lifestyle. Statistics refer to stool samples only. Average relative abundance is computed on positive samples only. Raw data in **Supplementary Data 2**.

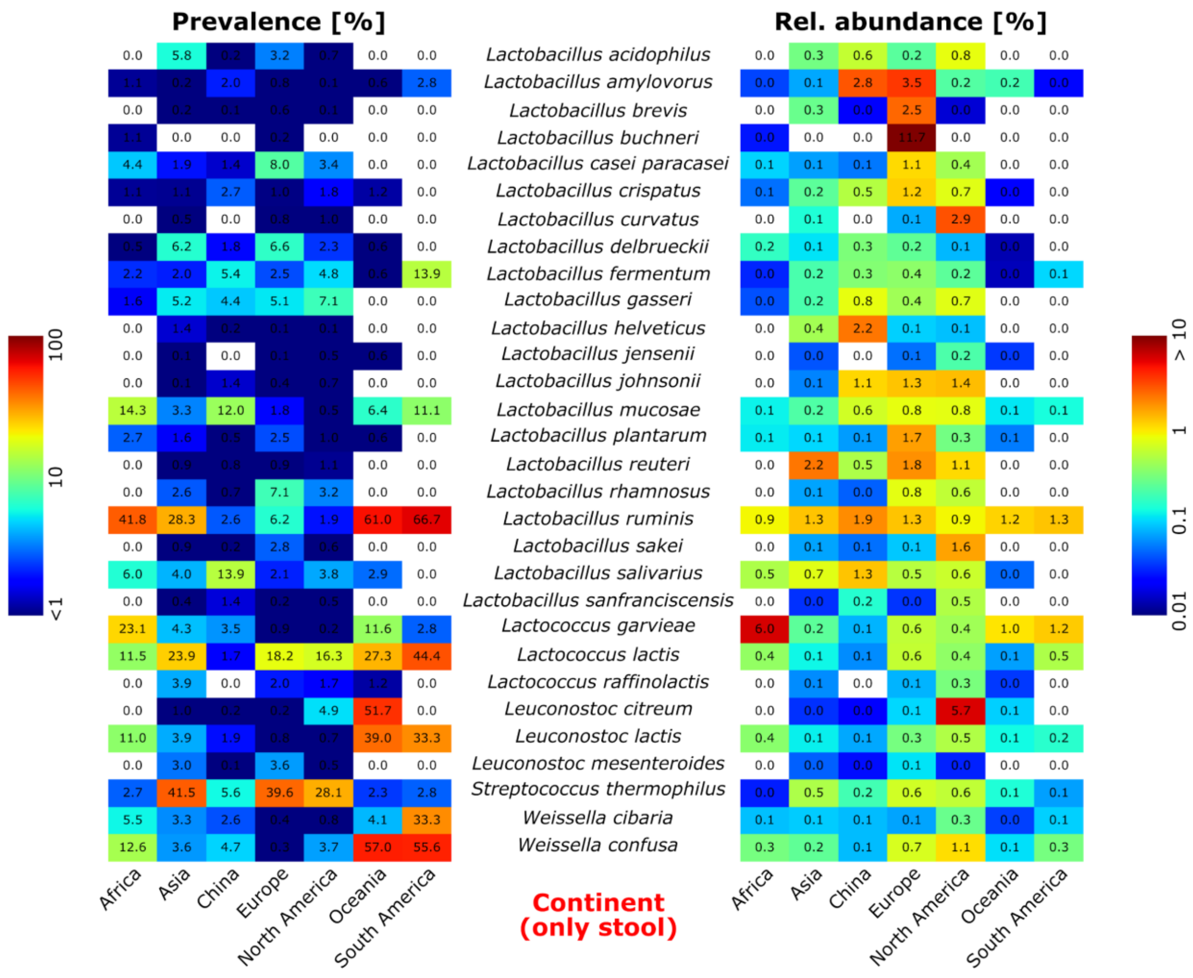

Supplementary Figure 4. Average prevalence and relative abundance of LAB species from the human microbiome stratified by continent. Statistics refer to stool samples only. Average relative abundance is computed on positive samples only. Raw data in **Supplementary Data 2**.

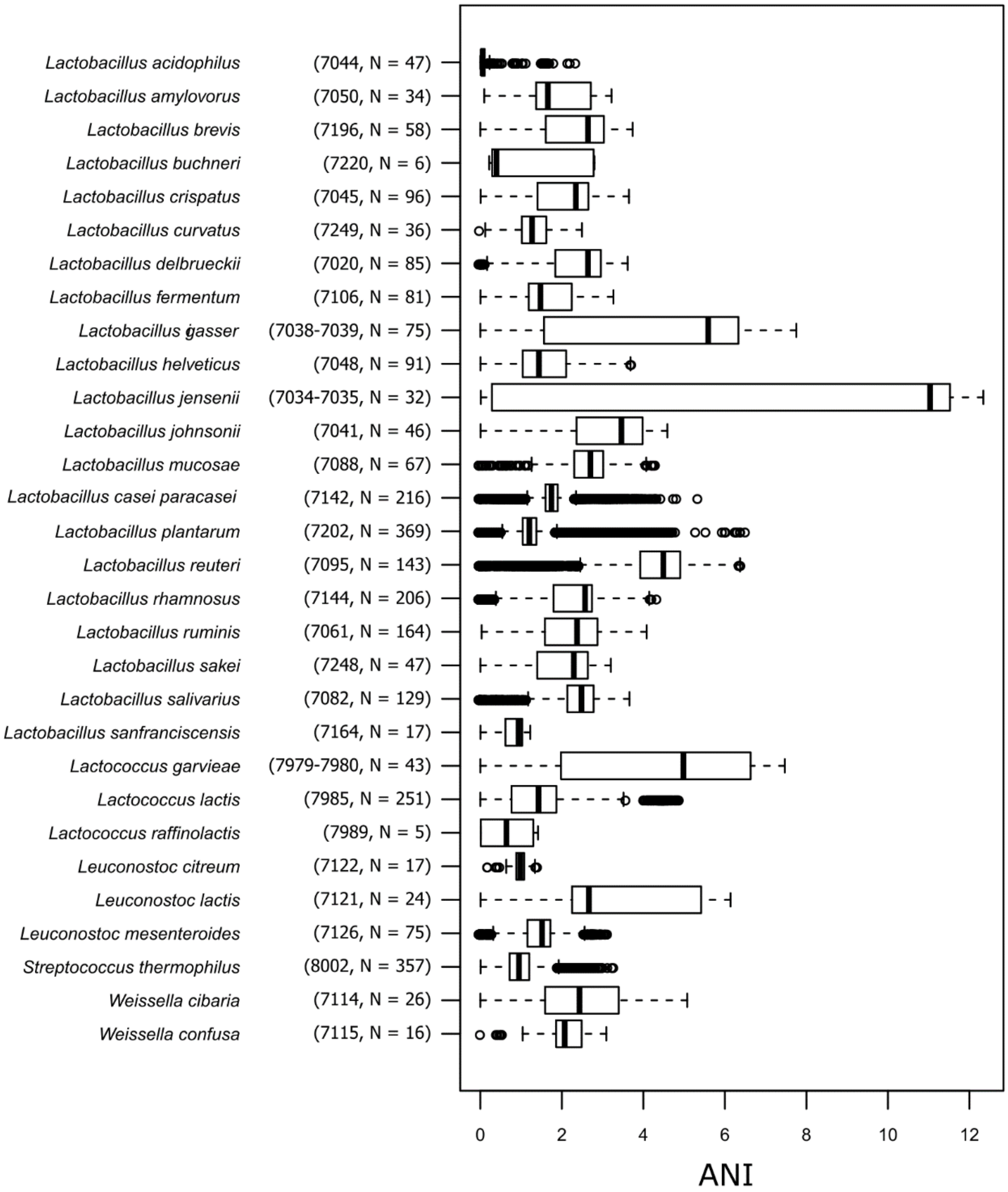

Supplementary Figure 5. Average nucleotide identity (ANI) for the 30 selected LAB species on the set of reference genomes and MAGs. The ANI is computed using FastANI [1] and excluding genomes having completeness < 80%. Numbers in parenthesis represent the SGB ID and the sample size. Three species (i.e., *Lb. gasseri*, *Lb. jensenii*, and *L. garvieae*) span two SGBs and are identified with two numeric identifiers. The lower and upper hinges correspond to the first and third quartiles (i.e., the 25th and 75th percentiles). The line inside the box represents the median value. The upper whisker extends from the hinge to the largest value no further than 1.5 * IQR from the hinge (where IQR is the inter-quartile range, or distance between the first and third quartiles). The lower whisker extends from the hinge to the smallest value at most 1.5 * IQR of the hinge. Points beyond the end of the whiskers are outliers and plotted individually.

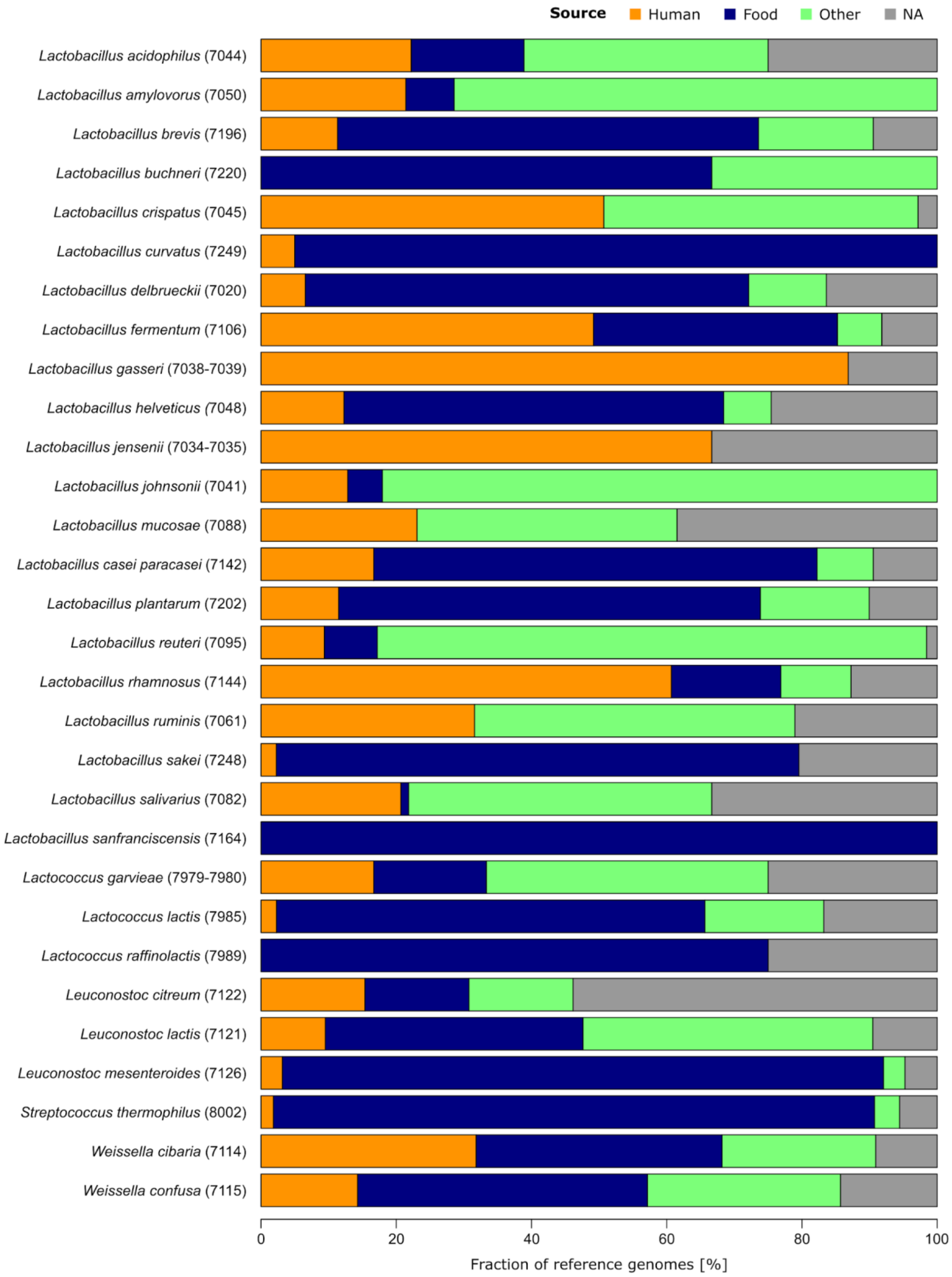

Supplementary Figure 6. Fraction of reference genomes per source type for the 30 selected LAB species. The same plot grouped at genus-level is reported in **Figure 2C**.

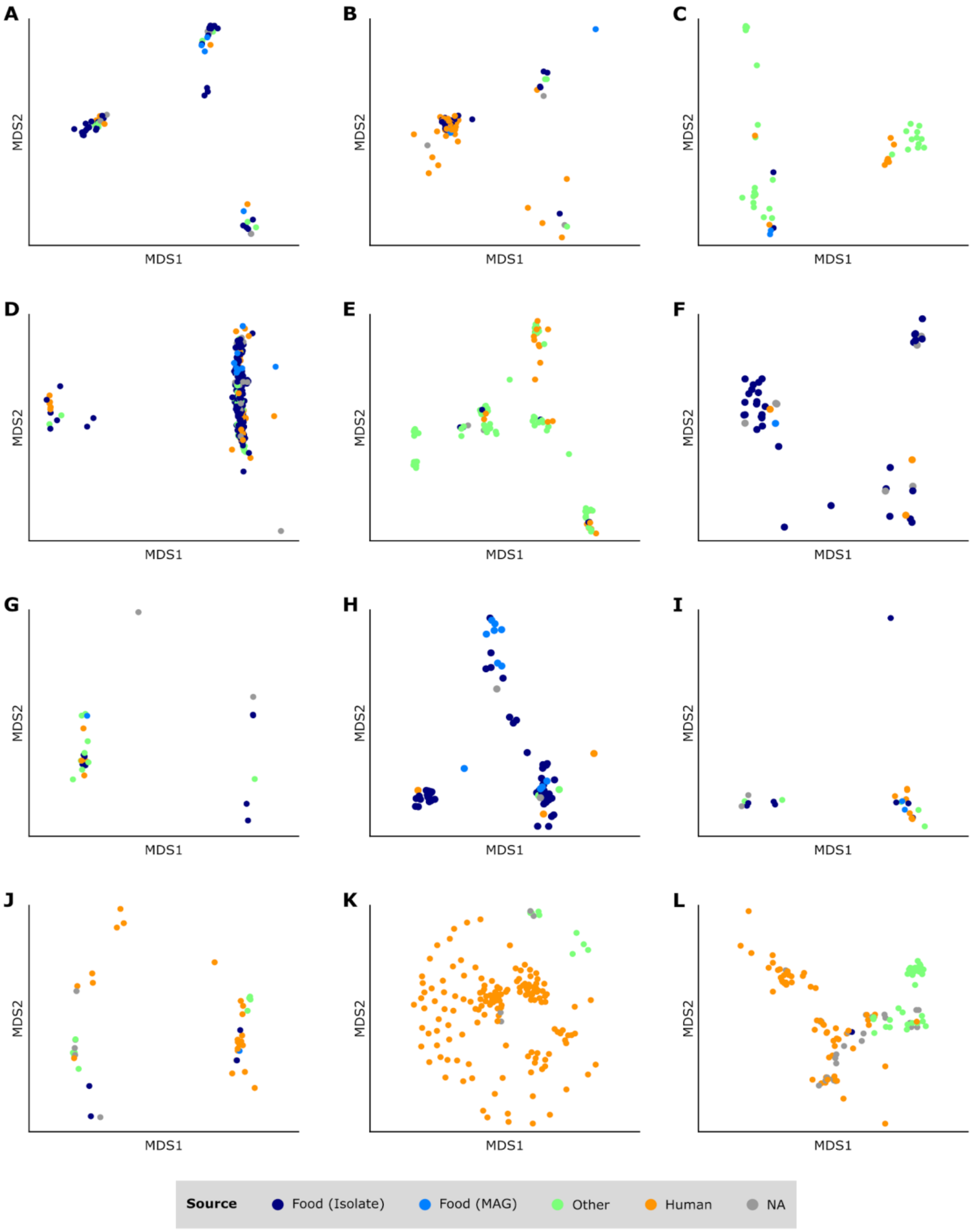

A
B
C
D
E
F
G
H
I
J
K
L
MDS1
MDS2
Source
Food (Isolate)
Food (MAG)
Other
Human
NA

Supplementary Figure 7. Comparative genomic analysis of relevant LAB species. Results refer to **A)** *Lb. brevis* (N = 58); **B)** *Lb. fermentum* (N = 81); **C)** *Lb. johnsonii* (N = 46); **D)** *Lb. plantarum* (N = 369); **E)** *Lb. reuteri* (N = 143); **F)** *Lb. sakei* (N = 47); **G)** *Leuconostoc lactis* (N = 24); **H)** *Leuconostoc mesenteroides* (N = 75); **I)** *W. cibaria* (N = 26); **J)** *L. garvieae* (N = 43); **K)** *Lb. ruminis* (N = 164); and **L)** *Lb. salivarius* (N = 129). **A)-I)** Species having occurrence of multiple subspecies into the same SGB with genomes from both food and human gut sources; **J)** *L. garvieae* is spread into two different SGBs; and **K)**-**L)** the typical non-food origin species *Lb. ruminis* and *Lb. salivarius* exhibit genomes extracted from the gut that are distinct from genomes isolated from other environments and animal microbiomes, suggesting long-term adaptation of these species to the human gut. Multidimensional scaling (MDS) on average nucleotide identity (ANI) distance is colored with source information. Plots for additional species are reported in **Figure 3** and **Figure 4**.

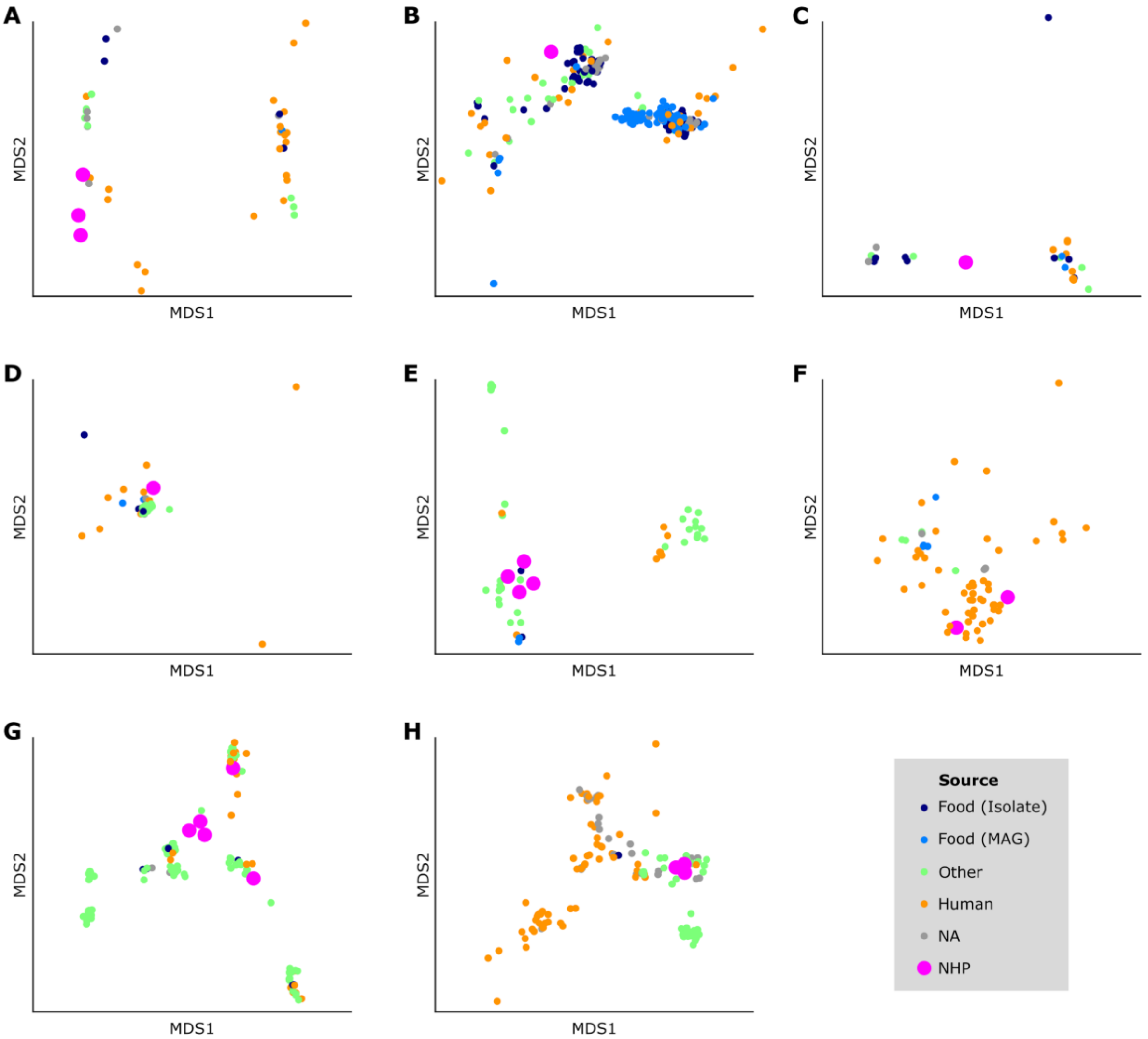

Supplementary Figure 8. Comparative genomic analysis of the LAB species reconstructed from NHP metagenomes that overlap with MAGs extracted from the human gut. Results refer to **A)** *Lc. garvieae* (N = 3 NHP MAGs); **B)** *Lc. lactis* (N = 1); **C)** *Weissella cibaria* (N = 1); **D)** *Lb. acidophilus* (N = 1); **E)** *Lb. johnsonii* (N = 4); **F)** *Lb. mucosae* (N = 2); **G)** *Lb. reuteri* (N = 5); and **H)** *Lb. salivarius* (N = 3).. NHP MAGs were retrieved from **A)-C)** wild NHPs and **D)-H)** NHPs living in captivity. Multidimensional scaling (MDS) on average nucleotide identity (ANI) distance is coloured with source information.

Supplementary References

1. Jain, C., Rodriguez-R, L. M., Phillippy, A. M., Konstantinidis, K. T. & Aluru, S. High throughput ANI analysis of 90K prokaryotic genomes reveals clear species boundaries. *Nat. Commun.* **9**, 5114 (2018).
