# Supplementary material for: Large-scale genome-wide analysis links lactic acid bacteria from food with the gut microbiome
Source: Nat Commun. 2020 May 25;11:2610. doi: 10.1038/s41467-020-16438-8 (PMC7248083; doi:10.1038/s41467-020-16438-8)
Supplement: Supplementary file 3 — Description of Additional Supplementary Files [file 41467_2020_16438_MOESM3_ESM.docx]

**Large-scale genome-wide analysis of lactic acid bacteria bridges food and gut microbiome**

Pasolli *et al.*

**Supplementary Data**

**Supplementary Data 1.** Description of the food metagenomes collected in this study.

**Supplementary Data 2.** Taxonomic profiles generated with MetaPhlAn2 with curated metadata of all metagenomic samples from human microbiomes considered in this paper.

**Supplementary Data 3.** Average prevalence and relative abundance of the 30 LAB species across the 303 food metagenomes estimated with MetaPhlAn2.

**Supplementary Data 4.** P-values associated with the Fisher’s test (after FDR correction) applied on the presence/absence of the 30 LAB species determined by MetaPhlAn2 spanning age, body site, continent, and westernized lifestyle categories.

**Supplementary Data 5.** Description of the MAGs extracted in this study from food metagenomes with their assigned SGB and estimated taxonomy.

**Supplementary Data 6.** Summary of the SGBs retrieved from food metagenomes in this study, and their overlap with SGBs extracted from human metagenomes.

**Supplementary Data 7.** Source type for the reference genomes associated with the LAB species considered in this study.

**Supplementary Data 8**. Description of the MAGs extracted from non-human primate metagenomes with their assigned SGB and estimated taxonomy. pSGBs (primate SGBs) identify newly assembled SGBs from NHPs metagenomes only.

**Supplementary Data 9**. List of sugar metabolism genes found to be differently prevalent (p < 0.05) between food and human gut genomes in *S. thermophilus* and *Lc. lactis*.

**Supplementary Data 10.** Number of markers per species used to build the phylogenies through PhyloPhlAn.
