# Supplementary material for: Large-scale genome-wide analysis links lactic acid bacteria from food with the gut microbiome
Source: Nat Commun. 2020 May 25;11:2610. doi: 10.1038/s41467-020-16438-8 (PMC7248083; doi:10.1038/s41467-020-16438-8)
Supplement: Supplementary file 14 — Reporting Summary [file 41467_2020_16438_MOESM14_ESM.pdf]

nature research

Corresponding author(s): Prof. Danilo Ercolini

Last updated by author(s): Apr 27, 2020

Reporting Summary

Nature Research wishes to improve the reproducibility of the work that we publish. This form provides structure for consistency and transparency in reporting. For further information on Nature Research policies, see Authors & Referees and the Editorial Policy Checklist .

Statistics

For all statistical analyses, confirm that the following items are present in the figure legend, table legend, main text, or Methods section.

n/a	Confirmed	
☐	☒	The exact sample size (*n*) for each experimental group/condition, given as a discrete number and unit of measurement
☐	☒	A statement on whether measurements were taken from distinct samples or whether the same sample was measured repeatedly
☐	☒	The statistical test(s) used AND whether they are one- or two-sided<br>*Only common tests should be described solely by name; describe more complex techniques in the Methods section.*
☐	☒	A description of all covariates tested
☐	☒	A description of any assumptions or corrections, such as tests of normality and adjustment for multiple comparisons
☐	☒	A full description of the statistical parameters including central tendency (e.g. means) or other basic estimates (e.g. regression coefficient) AND variation (e.g. standard deviation) or associated estimates of uncertainty (e.g. confidence intervals)
☐	☒	For null hypothesis testing, the test statistic (e.g. *F*, *t*, *r*) with confidence intervals, effect sizes, degrees of freedom and *P* value noted<br>*Give P values as exact values whenever suitable.*
☒	☐	For Bayesian analysis, information on the choice of priors and Markov chain Monte Carlo settings
☒	☐	For hierarchical and complex designs, identification of the appropriate level for tests and full reporting of outcomes
☒	☐	Estimates of effect sizes (e.g. Cohen's *d*, Pearson's *r*), indicating how they were calculated

Our web collection on statistics for biologists contains articles on many of the points above.

Software and code

Policy information about availability of computer code

Data collection	The raw data for the food metagenomes are available in NCBI-SRA under the BioProjects PRJEB6952, PRJEB15423, PRJEB15432, PRJEB20873, PRJEB32768, PRJEB35321, PRJNA286900, PRJNA430402, PRJNA482503, PRJNA603575, and in MG-RAST under the Project mgp3362.
Data analysis	All data analysis has been performed with open source software as comprehensively described in the methods of the paper. This includes MetaPhlAn2 (v. 2.0); metaSPAdes (v. 3.10.1); IDBA-UD (v. 1.1.3); MetaBAT2 (v. 2.12.1); CheckM (v. 1.0.7); Mash (v. 2.0); PhyloPhlAn (v. 3.0); GraPhlAn (v. 1.0); Prokka (v. 1.12); Roary (v. 3.11)

For manuscripts utilizing custom algorithms or software that are central to the research but not yet described in published literature, software must be made available to editors/reviewers. We strongly encourage code deposition in a community repository (e.g. GitHub). See the Nature Research guidelines for submitting code & software for further information.

Data

Policy information about availability of data

All manuscripts must include a data availability statement. This statement should provide the following information, where applicable:

- Accession codes, unique identifiers, or web links for publicly available datasets
- A list of figures that have associated raw data
- A description of any restrictions on data availability

The raw data for the food metagenomes are available in NCBI-SRA under the BioProjects PRJEB6952 [https://www.ncbi.nlm.nih.gov/bioproject/PRJEB6952], PRJEB15423 [https://www.ncbi.nlm.nih.gov/bioproject/PRJEB15423], PRJEB15432 [https://www.ncbi.nlm.nih.gov/bioproject/PRJEB15432], PRJEB20873 [https://www.ncbi.nlm.nih.gov/bioproject/PRJEB20873], PRJEB32768 [https://www.ncbi.nlm.nih.gov/bioproject/PRJEB32768], PRJEB35321 [https://www.ncbi.nlm.nih.gov/bioproject/PRJEB35321], PRJNA286900 [https://www.ncbi.nlm.nih.gov/bioproject/PRJNA286900], PRJNA430402 [https://www.ncbi.nlm.nih.gov/bioproject/PRJNA430402], PRJNA482503 [https://www.ncbi.nlm.nih.gov/bioproject/PRJNA482503], PRJNA603575 [https://www.ncbi.nlm.nih.gov/bioproject/PRJNA603575], and in MG-RAST under the Project mgp3362 [https://www.mg-rast.org/linkin.cgi?project=mgp3362].

The taxonomic profiles with associated metadata from the human metagenomes are available in the curatedMetagenomicData package 15. The MAGs from human metagenomes are available at http://segatalab.cibio.unitn.it/data/Pasolli_et_al.html. The newly reconstructed MAGs from food metagenomes are available at http://www.tfm.unina.it/DATA001-2020-Pasolli.

Field-specific reporting

Please select the one below that is the best fit for your research. If you are not sure, read the appropriate sections before making your selection.

- [x] Life sciences
- [ ] Behavioural & social sciences
- [ ] Ecological, evolutionary & environmental sciences

For a reference copy of the document with all sections, see nature.com/documents/nr-reporting-summary-flat.pdf

Life sciences study design

All studies must disclose on these points even when the disclosure is negative.

Sample size	This is a meta-analysis of 9445 publicly available shotgun human metagenomes that were previously anlyzed in Pasolli et al, "Extensive Unexplored Human Microbiome Diversity Revealed by Over 150,000 Genomes from Metagenomes Spanning Age, Geography, and Lifestyle", Cell 2019, in addition to 303 food metagenomes coming from 11 datasets collected in this study. This represents so far the largest meta-analysis that combines human and food shotgun metagenomes. Details on the sample size of each dataset are reported in the paper.
Data exclusions	All the metagenomes associated with the selected datasets are analyzed in this meta-analysis. Comparative genomics is done by excluding low quality genomes, i.e. with a completeness < 50% or contamination > 5% as estimated by CheckM.
Replication	This is a meta-analysis on a large collection of metagenomes and no replication is required.
Randomization	This is a meta-analysis on a large collection of metagenomes and no randomization is required.
Blinding	This is a meta-analysis on a large collection of metagenomes and no blinding is required.

Reporting for specific materials, systems and methods

We require information from authors about some types of materials, experimental systems and methods used in many studies. Here, indicate whether each material, system or method listed is relevant to your study. If you are not sure if a list item applies to your research, read the appropriate section before selecting a response.

Materials & experimental systems

n/a	Involved in the study	
x		Antibodies
x		Eukaryotic cell lines
x		Palaeontology
x		Animals and other organisms
x		Human research participants
x		Clinical data

Methods

n/a	Involved in the study	
x		ChIP-seq
x		Flow cytometry
x		MRI-based neuroimaging
